# The Reconciliation of Multiple Conflicting Estimates: Entropy-Based and Axiomatic Approaches

**DOI:** 10.3390/e20110815

**Published:** 2018-10-23

**Authors:** João F. D. Rodrigues, Michael L. Lahr

**Affiliations:** 1 Institute of Environmental Sciences CML, Leiden University, Einsteinweg 2, 2333 CC Leiden, The Netherlands; 2 Edward J. Bloustein School of Planning & Public Policy, Rutgers, The State University of New Jersey, 33 Livingston Avenue, New Brunswick, NJ 08901-1982, USA

**Keywords:** uncertainty modelling, economic accounts, conflicting estimates, entropy-based approach, axiomatix approach

## Abstract

When working with economic accounts it may occur that multiple estimates of a single datum exist, with different degrees of uncertainty or data quality. This paper addresses the problem of defining a method that can reconcile conflicting estimates, given best guess and uncertainty values. We proceeded from first principles, using two different routes. First, under an entropy-based approach, the data reconciliation problem is addressed as a particular case of a wider data balancing problem, and an alternative setting is found in which the multiple estimates are replaced by a single one. Afterwards, under an axiomatic approach, a set of properties is defined, which characterizes the ideal data reconciliation method. Under both approaches, the conclusion is that the formula for the reconciliation of best guesses is a weighted arithmetic average, with the inverse of uncertainties as weights, and that the formula for the reconciliation of uncertainties is a harmonic average.

## 1. Introduction

With improvements in information technology, the world has become more unified and interconnected. Information is now typically shared quickly and easily from all over the globe, such that barriers formed by linguistic and geographic boundaries essentially have been torn down. This has enabled people from disparate cultures and backgrounds to share ideas and information. One outcome of this regime change has been a boosting of the perceived benefits of statistical information. While some benefits of such statistical information have been known since at least Quetelet’s (1835) tome on so-called “social physics” was published, today’s massive socio-economic statistical repositories in Europe, North America, and East Asia are enabling a data revolution of sorts. Indeed, the fields of data mining and data analytics are fast becoming important fields of academic study. Mirroring the rise of data availability and the nature of some of the data itself, the term “big data” has been coined [1] to refer to the extremely voluminous and complex data sets that require specialized processing application software to deal with them.

The most prominent stewards of socio-economic data are government statistical agencies, which focus on producing and disseminating data products secured via surveys (for example the American Community Survey), censuses (such as Japan’s 2015 Population Census), and administrative procedures (like information needed to get an academic promotion in Spain). As a result, data storage is now ubiquitously electronic, replicated offsite to guard against storage failure, and measured in petabytes. Electronic storage enables low-cost dissemination of data. It also facilitates the integration of records across disparate databases—for example, into a system of national accounts, which is what countries use to generate their estimates of gross domestic product, as suggested by the United Nations. Both lead to concerns about confidentiality of data and how it can be protected [2].

Our point in broaching the above is that data producers, disseminators, and users alike can run into the problem of having access to multiple estimates for a single quantity of interest. In the particular experience of the authors, which motivated the present study, these multiple estimates are a consequence of non-disclosure by a statistical office in order to ensure confidentiality. Hence we act as what Duncan et al. [2] called “data snooper”. For concreteness, in such instances we are interested in obtaining number of employees at county level from the U.S. Bureau of Statistics’s Quarterly Census of Employment and Wages (QCEW) data and U.S. Bureau of the Census’s County Business Patterns. In both datasets these figures are suppressed for selected sectors in some counties and even states. But information is provided for larger spatial and sectoral units, so it is possible to use this higher-level information to obtain multiple estimates of the quantities of interest. It is common for official statistical data to have a hierarchical structure so this problem is quite general. Garfinkel et al. [3] note that the increasing ability of data snoopers is making ever more data stewards reluctant to provide certain data products because they are finding it increasingly difficult to ensure confidentiality to the agents from whom they obtain the data. This is despite some use of noise as a disclosure limitation [4].

In this paper, we focus on the problem of combining such multiple estimates into a single value. In the case of economic accounts, Miller and Blair [5] (pp. 384–386) have called it “the reconciliation issue”. The reconciliation issue considered here should not be confused with the more general problem of data balancing, in which a set of multiple data points need to satisfy a set of constraints: That problem is addressed in other studies, such as Kruithof [6], Stone et al. [7], Byron [8], Van Der Ploeg [9], Lahr and Mesnard [10], Chen [11]. General solutions to confidentiality disclosure or data censoring issues are provided by [12,13]. Herein we set out to assist current and future data snoopers and miners, by identifying what a data reconciliation method should be from first principles when a fairly general formulation of the reconciliation constraints is possible.

In particular, we consider that the multiple estimates for a particular datum can be characterized by a best guess and uncertainty. If we interpret each estimate as a random variable with an underlying probability distribution, the best guess is the expected value and the uncertainty is standard deviation. In the case of multiple data sources, the conflict enabling the multiple estimates is self-evident. When numbers are published with some data censored and for which estimates can be obtained using partial information [14], the conflict can arise from a higher (or lower) hierarchical spatial or sectoral level (e.g., average employee number if the number of establishments is available). To the best of our knowledge no first-principle approach to this problem has yet been published, although more heuristic approaches can be found in Bourque et al. [15], Miernyk et al. [16], Jensen and McGaurr [17], Gerking [18], Gerking [19], Weale [20], Boomsma and Oosterhaven [21], Rassier et al. [22]. We tackle the same problem from two different angles.

Using concepts and techniques from Bayesian inference [23] and in particular the minimum cross-entropy method [24], we first address the problem of data reconciliation as a particular case of more general data balancing [25]. That is, we consider there are two or more initial estimates for a particular datum, but this datum is itself embedded in a set of constraints connecting it to other data that are potentially unbalanced. We look for simplifications of the general setting under which this original problem can be transformed into another balancing problem where the multiple estimates are replaced by a single one. We prove that, if the initial uncertainty estimates are close to one another, the data reconciliation method of best guesses is a weighted arithmetic average and the data reconciliation method of uncertainties is a harmonic average.

Afterwards we address the same problem from an axiomatic perspective, laying out the desirable properties of a data reconciliation method. Such an approach has roots in different fields, from table deflation [26] and supply-use transformations [27] to environmental responsibility [28]. It turns out that the canonical data reconciliation method, i.e., the one that satisfies all required properties, is none other than a suitable generalization of the entropy-based method as derived earlier. That generalization centers on the introduction of the number of previously combined priors and a ranking of estimates by their relative quality.

## 2. Entropy-Based Approach

### 2.1. Basic Concepts

Bayesian inference was first developed by Laplace [29] and later expanded by others, such as Jeffreys [30], Jaynes [31] and Jaynes [23]. According to the Bayesian paradigm, a probability is a degree of belief about the likelihood of an event, and should reflect all relevant available information about that event. According to Weise and Woger [32], if an empirical quantity is subject to measurement errors, it must be described by a random variable, whose expectation is the best guess and whose standard-deviation is the uncertainty estimate.

More formally, a *prior* datum $\theta_{i}$ is characterized by a probability distribution $\pi (q_{i})$, which expresses the degree of belief that the datum takes realization $q_{i}$. The *best guess* is $\mu_{i}=E\left[\theta_{i}\right]$ and the *uncertainty* is $\sigma_{i}=\sqrt{\mathrm{Var}[\theta_{i}]}$. When multiple data are considered, e.g., $\theta_{i}$ and $\theta_{j}$, it is necessary to introduce the *correlation* between them, $\rho_{ij}=\mathrm{Cov}\left[\theta_{i},\theta_{j}\right]/\sigma_{i}\sigma_{j}$. Rodrigues [33] further provides a series of rules to determine the properties of a strictly positive prior datum, using the maximum-entropy principle [34].

The type of data we are interested in are connected to one another through *accounting identities* of the form:

$$\begin{array}{cc} \theta_{0} & =\sum_{i=1}^{n}\theta_{i}, \end{array}$$

where $\theta_{0}$ is an *aggregate* datum and the $\theta_{i}$’s are *disaggregate* data. If the set of data is arranged in a vector θ of length $n_{T}$, the set of $n_{K}$ accounting identities can be defined through a concordance matrix G, where, for a given accounting identity *i*, $G_{ij}=1$ if $\theta_{j}$ is a disaggregate datum, $G_{ij}=-1$ if $\theta_{j}$ is an aggregate datum and $G_{ij}=0$ otherwise.

If the prior configuration is unbalanced, then Gθ≠0, where 0 is a vector of zeros. Rodrigues [25] derives an analytical solution and a series of approximations that, given a concordance matrix and prior configuration, provide a *posterior* configuration, t, such that Gt=0. The notational convention used here is that Greek letters refer to priors while Latin cognates will refer to posteriors, i.e., $m_{i}$, $s_{i}$ and $r_{ij}$ are, respectively, the best guess and uncertainty of $t_{i}$ and correlation between $t_{i}$ and $t_{j}$.

### 2.2. Problem Formulation

**Figure 1:**
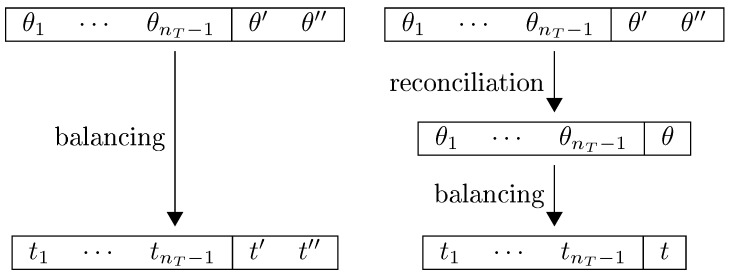
On the left-hand side balancing in a single step, with multiple initial estimates (priors) of the same datum, $\theta^{\prime }$ and $\theta^{\prime \prime }$, balanced to the same quantity (posterior), $t^{\prime }=t^{\prime \prime }$. On the right-hand side balancing in two steps: First the reconciliation procedures combines the multiple initial estimates (initial priors), $\theta^{\prime }$ and $\theta^{\prime \prime }$, into a final prior, θ; afterwards the full system is balanced, leading to posterior *t*. We impose that the result from both procedures is the same, $t=t^{\prime }=t^{\prime \prime }$.

We are now in position to formulate the data reconciliation problem. Given *initial priors* $\theta^{\prime }$ and $\theta^{\prime \prime }$ and a system with $n_{T}+1$ numerical data $\left\{\theta_{1},\ldots ,\theta_{n_{T}-1},\theta^{\prime },\theta^{\prime \prime }\right\}$, and $n_{K}+1$ accounting identities, where accounting identity $n_{K}+1$ takes the form $\theta^{\prime }=\theta^{\prime \prime }$, our goal is to determine the *final prior* θ, in a new system with $n_{T}$ numerical data, $\left\{\theta_{1},\ldots ,\theta_{n_{T}-1},\theta \right\}$ and the $n_{K}$ first accounting identities of the original system, in which the posteriors $\left\{t_{1},\ldots ,t_{n_{T}-1}\right\}$ are identical in both data balancing problems, and $t=t^{\prime }=t^{\prime \prime }$. Conceptually, we are approaching data reconciliation as a form of preliminary data balancing, as illustrated in Figure 1. The conflicting estimates are initial priors of the same datum, and the reconciled value is a final prior. Note the following notational convention: while other variables (and their properties) are denoted with subscripts, initial priors/posteriors (and their properties) are denoted with one (${}^{\prime }$) or two (${}^{\prime \prime }$) primes, and the final prior/posterior is denoted with neither subscripts nor primes.

Three situations emerge: Either the datum to be reconciled is only a disaggregate datum; it is only an aggregate datum; or it is both a disaggregate and an aggregate datum, in different accounting identities. We will deal with the three cases separately.

We now present simple systems to illustrate the three possible cases. As a benchmark consider a tabular system (i.e., with data organized in rows and columns) with no multiple estimates consisting of a 2×3 table A with row sums b and columns sums c. Furthermore, consider that the sum of both b and c is known as *d*. If i is a vector of ones of appropriate length, all vectors are in column format by default, and prime (${}^{\prime }$) adjoined to a matrix or vector denotes transpose, then the previous set of constraints means that:

$$\begin{array}{cc} Ai & =b;\\ A^{\prime }i & =c;\\ b^{\prime }i & =d;\\ c^{\prime }i & =d. \end{array}$$

The vectorized form of this system and the concordance table is presented in Table 1. In the baseline system there is a total of twelve variables (columns of the concordance matrix G) and seven constraints (rows thereof). The first six variables are disaggregate values (corresponding to the initial A matrix), the following five are mixed (row and column sums b and c), and the last one is an aggregate datum (*d*). The first two constraints (rows of G) are the row sums of A, the following three are its columns sums, and the last two are the sums of b and c. To understand how G is constructed let us consider the first constraint, which is the row sum of A. Formally, this is:

$$\begin{array}{cc} A_{11}+A_{12}+A_{13}-b_{1} & =0, \end{array}$$

hence in the first row of G the entries corresponding to the columns of $A_{11}$, $A_{12}$ and $A_{13}$ have 1s, the entry corresponding to the column of $b_{1}$ has −1 and all entries are zero.

We are now in position to formalize the three situations of multiple estimates of a single datum as variants of Table 1 in which an additional row and column has been added to G.

The case of disaggregate datum occurs if the datum for which multiple estimates exist is an interior point, which for concreteness we consider to be element $A_{23}$: The set of constraints is shown in Table 2. As an illustration of the case of there being two estimates of an aggregate datum consider it to be *d*: The set of constraints is shown in Table 3. Finally, consider as example of an element that is both aggregate and disaggregate that of $b_{1}$: The set of constraints is shown in Table 4.

It is perhaps instructive to describe how the reconciliation problems differ from the features of the baseline system. The three variants of the baseline are constructed by adding a single variable, the conflicting estimate, which by convenience is always appended to the original system. It is also necessary to add an extra constraint, connecting the two conflicting estimates. Finally, the baseline system is also changed so that in one of the original occurrences of the datum to be reconciled is the first conflicting estimate and the second occurrence is the other conflicting estimate.

Note that in this simple example there are only two constraints affecting each datum, but that naturally is not generally the case. The number of constraints per datum is arbitrary and can be either one or larger than two. An example of what this system might represent is employment count by region and sector, with an extra dimension being type of ownership (private or local, state, or federal government), as reported in the QCEW database.

### 2.3. From Balancing to Reconciliation

Rodrigues [25] shows that if the posterior configuration is balanced, then its first- and second-moment constraints are:

(1) $$\begin{array}{cc} 0 & =Gm; \end{array}$$

(2) $$\begin{array}{cc} 0 & =\mathrm{diag}\left({GS|G|}^{\prime }\right), \end{array}$$

where m and S are the posterior best-guess vector and covariance matrix, and the latter is defined as $S=\hat{s}R\hat{s}$, where s is the vector of posterior uncertainties and R is the vector of posterior correlations, and $\hat{\;}$ denotes diagonal matrix. Likewise μ and Σ are the prior best guess vector and covariance matrix, and the latter is defined as $\Sigma =\hat{\sigma }P\hat{\sigma }$.

The analytical solution of the data-balancing problem is:

(3) $$\begin{array}{cc} {\tilde{S}}^{-1} & ={\tilde{\Sigma }}^{-1}+G^{\prime }\hat{\beta }\left|G\right|; \end{array}$$

(4) $$\begin{array}{cc} {\tilde{S}}^{-1}\tilde{m} & ={\tilde{\Sigma }}^{-1}\tilde{\mu }+G^{\prime }\alpha . \end{array}$$

Notice that Equations (3) and (4) contain symbols adjoined with $\tilde{\;}$ (which we refer to as Gaussian parameters) while Equations (1) and (2) do not. The connection between the Gaussian parameters and the corresponding observable quantities is described in Rodrigues [25]: When relative uncertainty, $\sigma_{j}/\mu_{j}$ or $s_{j}/m_{j}$, is low, then the Gaussian parameter and the observable are identical. When relative uncertainty is high, the best guess Gaussian parameter tends to −∞ and the uncertainty Gaussian parameter tends to *∞*, in such a way that if relative uncertainty is unitary, $-{\tilde{\mu }}_{j}/{\tilde{\sigma }}_{j}^{2}=1/\mu_{j}=1/\sigma_{j}$ and $-{\tilde{m}}_{j}/{\tilde{s}}_{j}^{2}=1/m_{j}=1/s_{j}$. There is no closed-form expression between observables and Gaussian parameters in the multivariate case.

If both the prior uncertainty of aggregate data and initial prior correlations are high, we obtain a simplified weighted least-squares (WLS) method in which the weights are prior uncertainties:

(5) $$\begin{array}{cc} m & =\mu +\hat{\sigma }G^{\prime }\alpha , \end{array}$$

and posterior correlations are set by considering that relative uncertainty is constant, s=m⊙σ⊘μ, where ⊙ and ⊘ are Hadamard (or entrywise) product and division, and the update takes place in small steps.

This WLS method is a generalization of the standard biproportional balancing method (RAS) for arbitrary structure and uncertainty data [25]. However, it is in a way too simple for the data reconciliation problem, because it keeps relative uncertainty constant. In the data reconciliation problem this assumption is untenable, whenever the relative uncertainty of the initial priors differs.

Thus, we now look for a simplification of the general solution (Equations (3) and (4)) that is still feasible and that allows both for best guess and uncertainty reconciliation. Let us consider that correlations change little from prior to posterior, so that only uncertainties are adjusted. Equations (3) and (4) become:

$$\begin{array}{cc} {\hat{s}}^{-1}R^{-1}s^{-1} & ={\hat{\sigma }}^{-1}P^{-1}\sigma^{-1}+G^{\prime }\beta ;\\ {\hat{s}}^{-1}R^{-1}{\hat{s}}^{-1}m & ={\hat{\sigma }}^{-1}P^{-1}{\hat{\sigma }}^{-1}\mu +G^{\prime }\alpha , \end{array}$$

where we dropped the $\tilde{\;}$, meaning that all variables are observables. If correlations are not adjusted, then R=P, and if variances change little s≃σ The previous expressions become:

$$\begin{array}{cc} s^{-1} & =\sigma^{-1}+P\hat{\sigma }G^{\prime }\beta ;\\ {\hat{s}}^{-1}m & ={\hat{\sigma }}^{-1}\mu +P\hat{\sigma }G^{\prime }\alpha . \end{array}$$

For convenience, consider now that a datum corresponding to entry (i,j) in the tabular matrix is $t_{ij}$, while the sums of row or column *i* is $t_{i}$, and the Lagrange parameters of a row sum or column sum are adjoined with superscript *R* or *C*. For a particular entry, the previous matrix equation reads:

$$\begin{array}{cc} \frac{1}{s_{ij}} & =\frac{1}{\sigma_{ij}}+\sigma_{i}^{R}\beta_{i}^{R}+\sigma_{j}^{C}\beta_{j}^{C}+\sum_{k\neq i,j}\left(\sigma_{ik}\beta_{k}^{C}+\sigma_{kj}\beta_{k}^{R}\right);\\ \frac{1}{s_{i}} & =\frac{1}{\sigma_{i}}+\sigma_{i}\left(\beta_{i}^{R}+\beta_{i}^{C}\right), \end{array}$$

where $\sigma_{i}^{R}=\sum_{j}\sigma_{ij}$ and $\sigma_{i}^{C}=\sum_{j}\sigma_{ji}$. If the adjustment from prior to posterior is small, then $\sigma_{i}^{R}≃\sigma_{i}^{C}≃\sigma_{i}$. If $\beta_{i}^{*}=\sigma_{i}\beta_{i}^{R}$, $\sigma_{i}\gg \sigma_{ij}$ and $\sigma_{i}\gg \sigma_{ji}$, then the previous expression matrix expressions simplify to:

(6) $$\begin{array}{cc} s^{-1} & =\sigma^{-1}+G^{\prime }\beta^{*}; \end{array}$$

(7) $$\begin{array}{cc} {\hat{s}}^{-1}m & ={\hat{\sigma }}^{-1}\mu +G^{\prime }\alpha^{*}, \end{array}$$

where the derivation of Equation (7) follows along identical lines to that of Equation (6). We now use these expressions to obtain a tentative solution of the data reconciliation problem, even though they were derived under rather strict assumptions.

### 2.4. A Tentative Solution

We now examine the implications of applying Equations (6) and (7) to different data reconciliation configurations as described in Section 2.2: multiple estimates of (a) an aggregate datum; (b) a disaggregate datum; and (c) a datum that is both aggregate and disaggregate. We shall see that the same expression applies to all these problems.

For clarity, the analysis is carried out using scalar expressions, and, for brevity, only to the case of two constraints per datum. The strategy of the proof is the same for all configurations: to derive constraints connecting prior and posterior in the original problem and in a modified problem in which there is only a single datum where originally there were the conflicting estimates.

#### 2.4.1. Aggregate Datum

Consider that there are two initial priors of a datum, $\theta_{0}^{\prime }$ and $\theta_{0}^{\prime \prime }$ and that the datum is involved in two accounting identities, the first summing over elements 1 to $n^{\prime }$ and the second summing over $n^{\prime }+1$ to $n^{\prime \prime }$:

$$\begin{array}{cc} t_{0}^{\prime } & =\sum_{i=1}^{n^{\prime }}t_{i};\\ t_{0}^{\prime \prime } & =\sum_{i=n^{\prime }+1}^{n^{\prime }+n^{\prime \prime }}t_{i};\\ t_{0}^{\prime } & =t_{0}^{\prime \prime }, \end{array}$$

where each $t_{i}$, for i>0, can be affected by other accounting identities. The Lagrange parameters associated with these three expressions in Equation (6) are denoted, respectively, by $\beta_{0}^{\prime }$, $\beta_{0}^{\prime \prime }$ and $\beta_{0}$. We wish to determine a final prior $\theta_{0}$, such that:

$$\begin{array}{cc} t_{0} & =\sum_{i=1}^{n^{\prime }}t_{i};\\ t_{0} & =\sum_{i=n^{\prime }+1}^{n^{\prime }+n^{\prime \prime }}t_{i}. \end{array}$$

Equation (6) reads, for the original problem:

$$\begin{array}{cc} \frac{1}{s_{i}} & =\frac{1}{\sigma_{i}}+\beta_{0}^{\prime }+\ldots \;\mathrm{if}\;1\leq i\leq n^{\prime };\\ \frac{1}{s_{i}} & =\frac{1}{\sigma_{i}}+\beta_{0}^{\prime \prime }+\ldots \;\mathrm{if}\;n^{\prime }+1\leq i\leq n^{\prime \prime };\\ \frac{1}{s_{0}^{\prime }} & =\frac{1}{\sigma_{0}^{\prime }}-\beta_{0}^{\prime }+\beta_{0};\\ \frac{1}{s_{0}^{\prime \prime }} & =\frac{1}{\sigma_{0}^{\prime \prime }}-\beta_{0}^{\prime \prime }-\beta_{0}, \end{array}$$

where *…* refers to other Lagrange parameters. And in the modified problem:

$$\begin{array}{cc} \frac{1}{s_{i}} & =\frac{1}{\sigma_{i}}+\beta_{0}^{\prime }+\ldots \;\mathrm{if}\;1\leq i\leq n^{\prime };\\ \frac{1}{s_{i}} & =\frac{1}{\sigma_{i}}+\beta_{0}^{\prime \prime }+\ldots \;\mathrm{if}\;n^{\prime }+1\leq i\leq n^{\prime \prime };\\ \frac{1}{s_{0}} & =\frac{1}{\sigma_{0}}-\beta_{0}^{\prime }-\beta_{0}^{\prime \prime }. \end{array}$$

Notice that for every datum i>0 the original and modified problem are identical. Because the posteriors of the aggregate datum are all identical, $s_{0}^{\prime }=s_{0}^{\prime \prime }=s_{0}$, we can write:

$$\begin{array}{cc} 2\frac{1}{s_{0}} & =2\left(\frac{1}{\sigma_{0}}-\beta_{0}^{\prime }-\beta_{0}^{\prime \prime }\right)\\ & =\frac{1}{\sigma_{0}^{\prime }}+\frac{1}{\sigma_{0}^{\prime \prime }}-\beta_{0}^{\prime }-\beta_{0}^{\prime \prime }+\beta_{0}-\beta_{0}. \end{array}$$

A similar expression can be obtained from Equation (7) for the final prior best guess, leading to the solution:

$$\begin{array}{cc} \frac{1}{\sigma_{0}} & =\frac{1}{2}\left(\frac{1}{\sigma_{0}^{\prime }}+\frac{1}{\sigma_{0}^{\prime \prime }}\right);\\ \frac{\mu_{0}}{\sigma_{0}} & =\frac{1}{2}\left(\frac{\mu_{0}^{\prime }}{\sigma_{0}^{\prime }}+\frac{\mu_{0}^{\prime \prime }}{\sigma_{0}^{\prime \prime }}\right). \end{array}$$

Thus, both the final prior of the absolute uncertainty, σ, and the relative uncertainty, σ/μ, are obtained as the harmonic average of the initial prior absolute and relative uncertainties.

#### 2.4.2. Disaggregate Datum

Consider now that there are two initial priors of an interior point, $\theta_{1}^{\prime }$ and $\theta_{1}^{\prime \prime }$, which is affected by two accounting identities, such that the posteriors satisfy:

$$\begin{array}{cc} t_{0}^{\prime } & =t_{1}^{\prime }+\sum_{i=2}^{n^{\prime }}t_{i};\\ t_{0}^{\prime \prime } & =t_{1}^{\prime \prime }+\sum_{i=n^{\prime }+1}^{n^{\prime }+n^{\prime \prime }}t_{i};\\ t_{1}^{\prime } & =t_{1}^{\prime \prime }. \end{array}$$

The Lagrange parameters associated with these three expressions are, as before, $\beta_{0}^{\prime }$, $\beta_{0}^{\prime \prime }$ and $\beta_{0}$. We wish to determine a final prior $\theta_{1}$, such that:

$$\begin{array}{cc} t_{0}^{\prime } & =t_{1}+\sum_{i=2}^{n^{\prime }}t_{i};\\ t_{0}^{\prime \prime } & =t_{1}+\sum_{i=n^{\prime }+1}^{n^{\prime }+n^{\prime \prime }}t_{i}. \end{array}$$

Equation (6) reads, for the original problem:

$$\begin{array}{cc} \frac{1}{s_{i}} & =\frac{1}{\sigma_{i}}+\beta_{0}^{\prime }+\ldots \;\mathrm{if}\;2\leq i\leq n^{\prime };\\ \frac{1}{s_{i}} & =\frac{1}{\sigma_{i}}+\beta_{0}^{\prime \prime }+\ldots \;\mathrm{if}\;n^{\prime }+1\leq i\leq n^{\prime \prime };\\ \frac{1}{s_{0}^{\prime }} & =\frac{1}{\sigma_{0}^{\prime }}-\beta_{0}^{\prime }+\ldots ;\\ \frac{1}{s_{0}^{\prime \prime }} & =\frac{1}{\sigma_{0}^{\prime \prime }}-\beta_{0}^{\prime \prime }+\ldots ;\\ \frac{1}{s_{1}^{\prime }} & =\frac{1}{\sigma_{1}^{\prime }}+\beta_{0}^{\prime }+\beta_{1};\\ \frac{1}{s_{1}^{\prime \prime }} & =\frac{1}{\sigma_{1}^{\prime \prime }}+\beta_{0}^{\prime \prime }-\beta_{1}, \end{array}$$

and in the modified problem:

$$\begin{array}{cc} \frac{1}{s_{i}} & =\frac{1}{\sigma_{i}}+\beta_{0}^{\prime }+\ldots \;\mathrm{if}\;2\leq i\leq n^{\prime };\\ \frac{1}{s_{i}} & =\frac{1}{\sigma_{i}}+\beta_{0}^{\prime \prime }+\ldots \;\mathrm{if}\;n^{\prime }+1\leq i\leq n^{\prime \prime };\\ \frac{1}{s_{0}^{\prime }} & =\frac{1}{\sigma_{0}^{\prime }}-\beta_{0}^{\prime }+\ldots ;\\ \frac{1}{s_{0}^{\prime \prime }} & =\frac{1}{\sigma_{0}^{\prime \prime }}-\beta_{0}^{\prime \prime }+\ldots ;\\ \frac{1}{s_{1}} & =\frac{1}{\sigma_{1}}+\beta_{0}^{\prime }+\beta_{0}^{\prime \prime }. \end{array}$$

As before, the data for which there are no conflicting estimates ($t_{0}^{\prime }$, $t_{0}^{\prime \prime }$ and $t_{i}$ with i>1) are subject to the same set of constraints in the original and in the modified problem. Because the posteriors of the disaggregate datum are all identical, $s_{1}^{\prime }=s_{1}^{\prime \prime }=s_{1}$, we can write:

$$\begin{array}{cc} 2\frac{1}{s_{1}} & =2\left(\frac{1}{\sigma_{1}}+\beta_{0}^{\prime }+\beta_{0}^{\prime \prime }\right)\\ & =\frac{1}{\sigma_{1}^{\prime }}+\frac{1}{\sigma_{1}^{\prime \prime }}+\beta_{0}^{\prime }+\beta_{0}^{\prime \prime }+\beta_{1}-\beta_{1}. \end{array}$$

At this stage it becomes clear that we will encounter exactly the same solution as in the case of an aggregate datum:

$$\begin{array}{cc} \frac{1}{\sigma_{1}} & =\frac{1}{2}\left(\frac{1}{\sigma_{1}^{\prime }}+\frac{1}{\sigma_{1}^{\prime \prime }}\right);\\ \frac{\mu_{1}}{\sigma_{1}} & =\frac{1}{2}\left(\frac{\mu_{1}^{\prime }}{\sigma_{1}^{\prime }}+\frac{\mu_{1}^{\prime \prime }}{\sigma_{1}^{\prime \prime }}\right). \end{array}$$

#### 2.4.3. Mixed Datum

Consider now that there are two initial priors, $\theta_{1}^{\prime }$ and $\theta_{1}^{\prime \prime }$, of a datum that is both aggregate and disaggregate, in different accounting identities, and whose posteriors satisfy:

$$\begin{array}{cc} t_{0} & =t_{1}^{\prime }+\sum_{i=2}^{n^{\prime }}t_{i};\\ t_{1}^{\prime \prime } & =\sum_{i=n^{\prime }+1}^{n^{\prime \prime }}t_{i};\\ t_{1}^{\prime } & =t_{1}^{\prime \prime }. \end{array}$$

As before the Lagrange parameters are denoted as $\beta_{0}^{\prime }$, $\beta_{0}^{\prime \prime }$ and $\beta_{1}$. We wish to determine a final prior $\theta_{1}$, such that:

$$\begin{array}{cc} t_{0} & =t_{1}+\sum_{i=2}^{n^{\prime }}t_{i};\\ t_{1} & =\sum_{i=n^{\prime }+1}^{n^{\prime \prime }}t_{i}. \end{array}$$

Equation (6) reads, for the original problem:

$$\begin{array}{cc} \frac{1}{s_{i}} & =\frac{1}{\sigma_{i}}+\beta_{0}^{\prime }+\ldots \;\mathrm{if}\;2\leq i\leq n^{\prime };\\ \frac{1}{s_{i}} & =\frac{1}{\sigma_{i}}+\beta_{0}^{\prime \prime }+\ldots \;\mathrm{if}\;n^{\prime }+1\leq i\leq n^{\prime \prime };\\ \frac{1}{s_{0}} & =\frac{1}{\sigma_{0}}-\beta_{0}^{\prime }+\ldots ;\\ \frac{1}{s_{1}^{\prime }} & =\frac{1}{\sigma_{1}^{\prime }}+\beta_{0}^{\prime }+\beta_{1};\\ \frac{1}{s_{1}^{\prime \prime }} & =\frac{1}{\sigma_{1}^{\prime \prime }}-\beta_{0}^{\prime \prime }-\beta_{1}, \end{array}$$

and in the modified problem:

$$\begin{array}{cc} \frac{1}{s_{i}} & =\frac{1}{\sigma_{i}}+\beta_{0}^{\prime }+\ldots \;\mathrm{if}\;2\leq i\leq n^{\prime };\\ \frac{1}{s_{i}} & =\frac{1}{\sigma_{i}}+\beta_{0}^{\prime \prime }+\ldots \;\mathrm{if}\;n^{\prime }+1\leq i\leq n^{\prime \prime };\\ \frac{1}{s_{0}} & =\frac{1}{\sigma_{0}}-\beta_{0}^{\prime }+\ldots ;\\ \frac{1}{s_{1}} & =\frac{1}{\sigma_{1}}+\beta_{0}^{\prime }-\beta_{0}^{\prime \prime }. \end{array}$$

As has become routine, for datum 0 and for every datum i>1 the original and modified problem are identical. Because $s_{1}^{\prime }=s_{1}^{\prime \prime }=s_{1}$, we can write:

$$\begin{array}{cc} 2\frac{1}{s_{1}} & =2\left(\frac{1}{\sigma_{1}}+\beta_{0}^{\prime }-\beta_{0}^{\prime \prime }\right)\\ & =\frac{1}{\sigma_{1}^{\prime }}+\frac{1}{\sigma_{1}^{\prime \prime }}+\beta_{0}^{\prime }-\beta_{0}^{\prime \prime }+\beta_{1}-\beta_{1}. \end{array}$$

Thus, it is clear that the solution is again identical.

## 3. Axiomatic Approach

### 3.1. Axiomatic Formulation

In Section 2 we obtained a data reconciliation algorithm from first principles, as an operation of data balancing under a particular structure. However, we can also reason about the data reconciliation algorithm in terms of its properties, i.e., we will not determine what it is, but what it ought to be.

If $\theta^{\prime }$ and $\theta^{\prime \prime }$ are two initial priors, the data reconciliation algorithm is a function f(·) that generates a final prior $\theta =f(\theta^{\prime },\theta^{\prime \prime })$, where each prior θ is characterized by a best guess, μ, an absolute uncertainty, σ, and a relative uncertainty, u=σ/μ, which can take values in the range 0≤u≤1. Let $x_{\min}=\min\left\{x^{\prime },x^{\prime \prime }\right\}$ and $x_{\max}=\max\left\{x^{\prime },x^{\prime \prime }\right\}$, where *x* can be μ, σ or *u*.

We now propose a series of properties that define the data reconciliation method.

**Property** **1** (Lower and upper bounds)**.** The parameters of the final prior lie within the range set by the parameters of the initial priors, $\mu_{\min}\leq \mu \leq \mu_{\max}$, $\sigma_{\min}\leq \sigma \leq \sigma_{\max}$ and $u_{\min}\leq u\leq u_{\max}$.

**Property** **2** (Commutativity)**.** The order in which the initial priors are combined does not matter, $f\left(\theta^{\prime },\theta^{\prime \prime }\right)=f\left(\theta^{\prime \prime },\theta^{\prime }\right)$.

**Property** **3** (Associativity)**.** Several initial priors can be combined and the resulting final prior is invariant to the order of reconciliation, $f\left(\theta^{\prime },f\left(\theta^{\prime \prime },\theta^{\prime \prime \prime }\right)\right)=f\left(f\left(\theta^{\prime },\theta^{\prime \prime }\right),\theta^{\prime \prime \prime }\right)$.

**Property** **4** (Identity)**.** If the initial prior best guesses are identical, $\mu^{\prime }=\mu^{\prime \prime }$ then the final prior best guess is identical, $\mu =\mu^{\prime }=\mu^{\prime \prime }$. If the initial prior uncertainties are identical, $\sigma^{\prime }=\sigma^{\prime \prime }$ then the final prior uncertainty is identical, $\sigma =\sigma^{\prime }=\sigma^{\prime \prime }$.

**Property** **5** (Monotonicity)**.** *The relative adjustment from initial to final prior increases with the relative magnitude of initial uncertainty:*

(8) $$\begin{array}{cc} \frac{\mu -\mu^{\prime }}{\mu^{\prime \prime }-\mu } & =g\left(\frac{\sigma^{\prime }}{\sigma^{\prime \prime }}\right); \end{array}$$

(9) $$\begin{array}{cc} \frac{\sigma -\sigma^{\prime }}{\sigma^{\prime \prime }-\sigma } & =h\left(\frac{\sigma^{\prime }}{\sigma^{\prime \prime }}\right). \end{array}$$

*where dg(x)/dx>0 and dh(x)/dx>0.*

**Property** **6** (Absorption)**.** If initial prior $\theta^{\prime }$ is known with minimal uncertainty, $u^{\prime }=0$, and $\theta^{\prime \prime }$ is not, $u^{\prime \prime }>0$, then the final prior is identical to the first initial prior, $f\left(\theta^{\prime },\theta^{\prime \prime }\right)=\theta^{\prime }$. If initial prior $\theta^{\prime }$ is known with maximal uncertainty, $u^{\prime }=1$, and $\theta^{\prime \prime }$ is not, $u^{\prime \prime }<1$, then the final prior is identical to the second initial prior, $f\left(\theta^{\prime },\theta^{\prime \prime }\right)=\theta^{\prime \prime }$.

We believe that these six properties are uncontroversial and self-explanatory. However, it turns out that the problem as formulated here has no solution, i.e., no formula can satisfy all of the above properties. We later overcome this hurdle by generalizing the problem formulation, to include two additional concepts: A hierarchy of data quality and the number of combined priors.

### 3.2. The Canonical Data Reconciliation Method

The properties outlined in Section 3.1 constrain the range of data reconciliation algorithms but do not define a unique solution. However, Equations (8) and (9) suggests how it may be possible to obtain a solution. Let us consider that g(x) and h(x) take the simple yet flexible form of $g\left(x\right)=ax^{b}$ and $h\left(x\right)=cx^{d}$.

The condition of identity (Property 4), in the case of $\mu^{\prime }=\mu^{\prime \prime }$ and $\sigma^{\prime }=\sigma^{\prime \prime }$ leads to the indeterminacy:

$$\begin{array}{cc} \frac{\mu -\mu^{\prime }}{\mu^{\prime \prime }-\mu } & =\frac{0}{0}. \end{array}$$

But if the limit is approached as $\mu^{\prime }=\mu -\delta$ and $\mu^{\prime \prime }=\mu +\delta$, when δ→0, then:

$$\begin{array}{cc} \frac{\mu -\mu^{\prime }}{\mu^{\prime \prime }-\mu } & =\frac{\delta }{\delta }=1. \end{array}$$

Thus, under the condition of identity, Equations (8) and (9) imply that:

$$\begin{array}{cc} 1 & =a1^{b};\\ 1 & =c1^{d}, \end{array}$$

so a=c=1. Let us further consider the simplest possible case b=d=1, so that g(·) and h(·) are the identity lines. Applying g(x)=x and h(x)=x to Equations (8) and (9) leads to:

$$\begin{array}{cc} \frac{\mu -\mu^{\prime }}{\sigma^{\prime }} & =\frac{\mu^{\prime \prime }-\mu }{\sigma^{\prime \prime }};\\ \frac{\sigma -\sigma^{\prime }}{\sigma^{\prime }} & =\frac{\sigma^{\prime \prime }-\sigma }{\sigma^{\prime \prime }}. \end{array}$$

Rearranging terms:

$$\begin{array}{cc} \mu \left(\frac{1}{\sigma^{\prime }}+\frac{1}{\sigma^{\prime \prime }}\right) & =\frac{\mu^{\prime }}{\sigma^{\prime }}+\frac{\mu^{\prime \prime }}{\sigma^{\prime \prime }};\\ \sigma \left(\frac{1}{\sigma^{\prime }}+\frac{1}{\sigma^{\prime \prime }}\right) & =\frac{\sigma^{\prime }}{\sigma^{\prime }}+\frac{\sigma^{\prime \prime }}{\sigma^{\prime \prime }}. \end{array}$$

Recalling that u=σ/μ we obtain the canonical data reconciliation method as:

(10) $$\begin{array}{cc} \mu & =\left(\frac{1/\sigma^{\prime }}{1/\sigma^{\prime }+1/\sigma^{\prime \prime }}\right)\mu^{\prime }+\left(\frac{1/\sigma^{\prime \prime }}{1/\sigma^{\prime }+1/\sigma^{\prime \prime }}\right)\mu^{\prime \prime }; \end{array}$$

(11) $$\begin{array}{cc} \frac{1}{\sigma } & =\frac{1}{2}\left(\frac{1}{\sigma^{\prime }}+\frac{1}{\sigma^{\prime \prime }}\right). \end{array}$$

Equation (10) can be be expressed in two other ways:

(12) $$\begin{array}{cc} \mu & =\frac{\sigma }{2}\left(\frac{\mu^{\prime }}{\sigma^{\prime }}+\frac{\mu^{\prime \prime }}{\sigma^{\prime \prime }}\right); \end{array}$$

(13) $$\begin{array}{cc} \frac{1}{u} & =\frac{1}{2}\left(\frac{1}{u^{\prime }}+\frac{1}{u^{\prime \prime }}\right). \end{array}$$

Thus, if the ratio of relative adjustment of best guesses and uncertainties is identical to the ratio of absolute uncertainties of the initial priors, the best-guess data reconciliation method is a weighted average, where the weights are proportional to the inverse of absolute uncertainty, and the absolute and relative uncertainty data reconciliation methods are harmonic averages.

Does this data reconciliation method satisfy the properties of Section 3.1? It is trivial to check that Properties 1, 2, 4 and 5 are satisfied. But this is not the case for Properties 3 and 6. In the following subsections we present suitable extensions of the canonical data reconciliation method to address these problems.

### 3.3. The Number of Combined Priors

The canonical data reconciliation method is not associative. The properties of $f(\theta^{\prime },f\left(\theta^{\prime \prime },\theta^{\prime \prime \prime }\right))$ are:

$$\begin{array}{cc} \frac{1}{u} & =\frac{1}{2}\left(\frac{1}{u^{\prime }}+\frac{1}{2}\left(\frac{1}{u^{\prime \prime }}+\frac{1}{u^{\prime \prime \prime }}\right)\right)=\frac{1}{2u^{\prime }}+\frac{1}{4u^{\prime \prime }}+\frac{1}{4u^{\prime \prime \prime }}\\ \frac{1}{\sigma } & =\frac{1}{2}\left(\frac{1}{\sigma^{\prime }}+\frac{1}{2}\left(\frac{1}{\sigma^{\prime \prime }}+\frac{1}{\sigma^{\prime \prime \prime }}\right)\right)=\frac{1}{2\sigma^{\prime }}+\frac{1}{4\sigma^{\prime \prime }}+\frac{1}{4\sigma^{\prime \prime \prime }}. \end{array}$$

While the properties of $f(\left(\theta^{\prime },\theta^{\prime \prime }\right),\theta^{\prime \prime \prime })$ are:

$$\begin{array}{cc} \frac{1}{u} & =\frac{1}{2}\left(\frac{1}{2}\left(\frac{1}{u^{\prime }}+\frac{1}{u^{\prime \prime }}\right)+\frac{1}{u^{\prime \prime \prime }}\right)=\frac{1}{4u^{\prime }}+\frac{1}{4u^{\prime \prime }}+\frac{1}{2u^{\prime \prime \prime }}\\ \frac{1}{\sigma } & =\frac{1}{2}\left(\frac{1}{2}\left(\frac{1}{\sigma^{\prime }}+\frac{1}{\sigma^{\prime \prime }}\right)+\frac{1}{\sigma^{\prime \prime \prime }}\right)=\frac{1}{4\sigma^{\prime }}+\frac{1}{4\sigma^{\prime \prime }}+\frac{1}{2\sigma^{\prime \prime \prime }}. \end{array}$$

Thus, $f\left(\theta^{\prime },f\left(\theta^{\prime \prime },\theta^{\prime \prime \prime }\right)\right)\neq f\left(\left(\theta^{\prime },\theta^{\prime \prime }\right),\theta^{\prime \prime \prime }\right)$. But upon some reflection, this result is in fact reasonable. The final prior is the combination of two initial priors with equal weights. If some of these initial priors are themselves a combination of other initial priors, this information has to be considered explicitly.

Let us introduce a new quantity, *n*, as the *number of combined priors*, so that now a prior θ is defined by a best guess, μ, an absolute uncertainty, σ, and *n*. Consider the following data reconciliation rule:

(14) $$\begin{array}{cc} \mu & =\left(\frac{n^{\prime }/\sigma^{\prime }}{n^{\prime }/\sigma^{\prime }+n^{\prime \prime }/\sigma^{\prime \prime }}\right)\mu^{\prime }+\left(\frac{n^{\prime \prime }/\sigma^{\prime \prime }}{n^{\prime }/\sigma^{\prime }+n^{\prime \prime }/\sigma^{\prime \prime }}\right)\mu^{\prime \prime }; \end{array}$$

(15) $$\begin{array}{cc} \frac{1}{\sigma } & =\frac{1}{n}\left(\frac{n^{\prime }}{\sigma^{\prime }}+\frac{n^{\prime \prime }}{\sigma^{\prime \prime }}\right); \end{array}$$

(16) $$\begin{array}{cc} n & =n^{\prime }+n^{\prime \prime }. \end{array}$$

As before, Equation (14) can be be expressed in two other ways:

(17) $$\begin{array}{cc} \mu & =\frac{\sigma }{n}\left(\frac{n^{\prime }}{\sigma^{\prime }}\mu^{\prime }+\frac{n^{\prime \prime }}{\sigma^{\prime \prime }}\mu^{\prime \prime }\right); \end{array}$$

(18) $$\begin{array}{cc} \frac{1}{u} & =\frac{1}{n}\left(\frac{n^{\prime }}{u^{\prime }}+\frac{n^{\prime \prime }}{u^{\prime \prime }}\right). \end{array}$$

This data reconcilation rule satisfies the first five properties of Section 3.1.

### 3.4. Ranking of Data Quality

The canonical data reconciliation method satisfies the absorption property of minimal uncertainty. If $\sigma^{\prime }=0$ and $\sigma^{\prime \prime }>0$, then:

$$\begin{array}{cc} \frac{1}{\sigma^{\prime }}+\frac{1}{\sigma^{\prime \prime }} & ≃\frac{1}{\sigma^{\prime }}, \end{array}$$

and Equations (10) and (11) become:

$$\begin{array}{cc} \mu & ≃\left(\frac{1/\sigma^{\prime }}{1/\sigma^{\prime }}\right)\mu^{\prime }+\left(\frac{1/\sigma^{\prime \prime }}{1/\sigma^{\prime }}\right)\mu^{\prime \prime }=\left(1\right)\mu^{\prime }+\left(0\right)\mu^{\prime \prime };\\ \sigma & ≃2\sigma^{\prime }=0, \end{array}$$

so $\mu =\mu^{\prime }$ and $\sigma =\sigma^{\prime }$. However, it does not satisfy the absorption property of maximal uncertainty. If $\sigma^{\prime }=\mu^{\prime }$ and $\sigma^{\prime \prime }<\mu^{\prime \prime }$, then $u^{\prime }=0$ and Equations (10), (11) and (13) become:

$$\begin{array}{cc} \mu & =\left(\mu^{\prime \prime }+\sigma^{\prime \prime }\right)\frac{\mu^{\prime }}{\mu^{\prime }+\sigma^{\prime \prime }};\\ \sigma & =2\sigma^{\prime \prime }\frac{\mu^{\prime }}{\mu^{\prime }+\sigma^{\prime \prime }};\\ u & =2\frac{u^{\prime \prime }}{1+u^{\prime \prime }}, \end{array}$$

and thus $\mu \neq \mu^{\prime \prime }$ and $\sigma \neq \sigma^{\prime \prime }$.

In order to ensure that the absorption of maximal uncertainty is satisfied, we use the concept of *data quality*, introduced in Rodrigues [25]. The idea is that, besides an uncertainty estimate, which formalizes quantitatively a degree of confidence in the accuracy of the best guess of a datum, it is also possible to formalize qualitatively a degree of confidence in the accuracy of a datum relative to others.

For the purpose of data balancing, Rodrigues [25] suggests that a datum that is considered to be of higher quality should be kept fixed while lower quality data are adjusted. The natural corollary, in the problem of data reconciliation, is to consider that when one wishes to combine two initial priors of differing levels of data quality, the prior of lower quality should be disregarded.

If a datum has unitary relative uncertainty, then it is maximally uninformative, and it is reasonable to disregard it. After all, a maximally uninformative prior should only be used if no better alternative is available. We therefore suggest that, if $\sigma^{\prime }=\mu^{\prime }$ and $\sigma^{\prime \prime }<\mu^{\prime \prime }$, then $\theta =\theta^{\prime \prime }$ directly, without using Equations (10) and (11).

### 3.5. Summary

We now present the expressions for the combination of *n* initial priors, $\theta_{i}$, with i=1,…,n into a single final prior θ. Addressing this problem requires the specification, for each prior, $\theta_{i}$, of its best guess, $\mu_{i}$, its absolute uncertainty, $\sigma_{i}$, and the number of previously combined priors, $n_{i}$.

If all relative uncertainties, $u_{i}=\sigma_{i}/\mu_{i}$, are in the range $0<u_{i}<1$, then the final prior properties are defined as:

(19) $$\begin{array}{cc} \mu & =\sum_{i=1}^{n}\left(\frac{n_{i}/\sigma_{i}}{n/\sigma }\right)\mu_{i}; \end{array}$$

(20) $$\begin{array}{cc} \frac{1}{\sigma } & =\frac{1}{n}\left(\sum_{i=1}^{n}\frac{n_{i}}{\sigma_{i}}\right); \end{array}$$

(21) $$\begin{array}{cc} n & =\sum_{i=1}^{n}n_{i}. \end{array}$$

Equation (19) can be expressed as:

(22) $$\begin{array}{cc} \frac{1}{u} & =\frac{1}{n}\sum_{i=1}^{n}\frac{n_{i}}{u_{i}}. \end{array}$$

If some initial priors have zero relative uncertainty, $u_{i}=0$, then all other initial priors should be disregarded. If some initial priors have unitary relative uncertainty, $u_{i}=1$, then it is they which should be disregarded.

**Figure 2:**
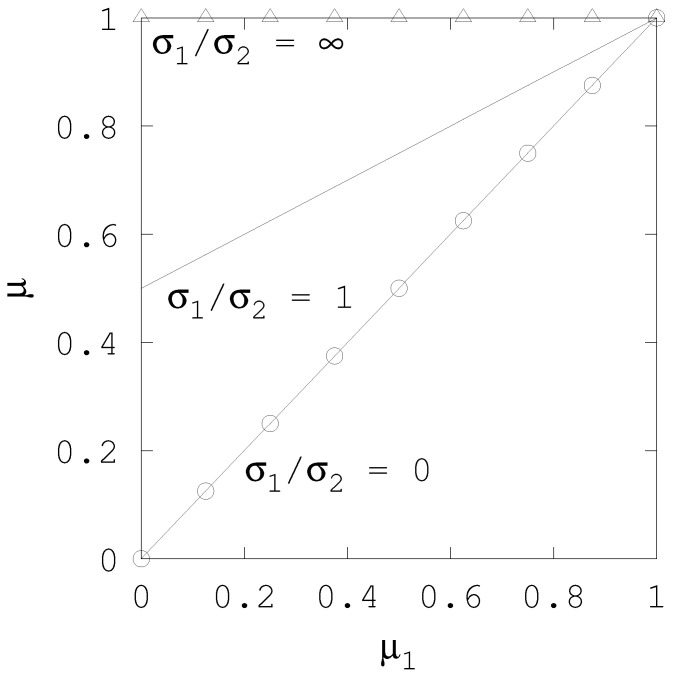
Best guess of the combined prior as a function of initial prior best guess, $\mu_{1}$, when $\mu_{2}=1$, for diferent values of $\sigma_{1}/\sigma_{2}$: Solid line (–) when $\sigma_{1}/\sigma_{2}=1$, solid line with circle markers (–○–) when $\sigma_{1}/\sigma_{2}=0$, and solid line with triangle markers (–△–) when $\sigma_{1}/\sigma_{2}=\infty$.

In Figure 2 we illustrate the behaviour of Equation (19), when n=2 and $n_{1}=n_{2}=\mu_{2}=1$. The plot shows different curves of the combined posterior best guess μ as a function of the prior best guess $\mu_{1}$, where each curve corresponds to a different ratio of uncertainties, $\sigma_{1}/\sigma_{2}$. When both uncertainties are identical, the posteriod best guess is the arithmetic average of the two prior best guesses. When the uncertainties differ, the best guess prior with the largest uncertainty contributes the least to combined best guess: in the limit case in which $\sigma_{1}\gg \sigma_{2}$ the prior $\mu_{1}$ is ignored and the posterior is similar to $\mu_{2}$; when $\sigma_{1}\ll \sigma_{2}$ the reverse occurs and $\mu ≃\mu_{1}$.

**Figure 3:**
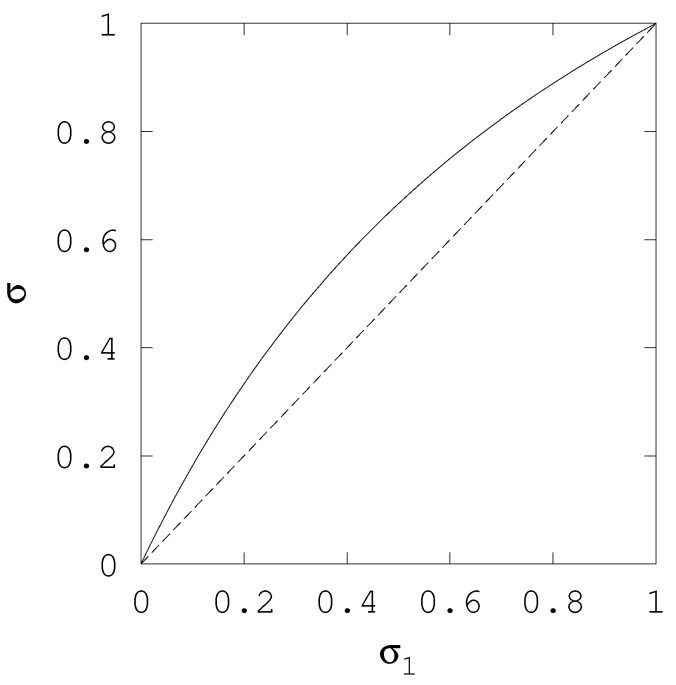
Solid line (–) and relative uncertainty, uncertainty of the final prior, σ, as a function of initial prior uncertainty, $\sigma_{1}$, when $\sigma_{2}=1$. Dashed line (--) is the identity line.

In turn, Figure 3 we illustrate the behaviour of Equation (20). We still consider that n=2 and $n_{1}=n_{2}$ but now no explicit assumption about best guesses is necessary. Instead, the uncertainty of the second variable is fixed, $\sigma_{2}=1$, and the curve shows the value of the combined posterior as a function of prior uncertainty $\sigma_{1}$. The figure describes an arc slightly above the diagonal line. When both prior uncertainties are identical ($\sigma_{1}=1$), then the posterior equals the priors, as expected. As $\sigma_{1}$ becomes smaller than $\sigma_{2}$ the combined prior becomes closer to $\sigma_{1}$ than to $\sigma_{2}$, but always larger, $\sigma >\sigma_{1}$, except in the limit case $\sigma_{1}\to 0$, in which case $\sigma \to \sigma_{1}$.

## 4. Conclusions and Discussion

Herein we investigated using two distinct pathways the problem of reconciling multiple conflicting estimates in the course of database development. We assume that the developer (data snooper) is tooled with a best guess and uncertainty for each of those conflicting estimates.

First, we apply a maximum-entropy Bayesian inference method, under the limiting condition that the adjustment from prior to posterior uncertainties is small. Second, we obtain a canonical data reconciliation method through an axiomatic approach that is as simple as possible but satisfied important qualitative properties. Each approach verifies the other.

The resulting formula for the best guess, Equation (19), is a weighted average showing that, as the count of conflicting priors underlying a particular prior rises, the value of that prior increases in importance in terms of obtaining a solution. We get a similar result with the inverse of the uncertainty, that is, the narrower the uncertainty of an estimate the more it contributes to the final solution. The resulting formula for the uncertainty, Equation (20), is a harmonic average where the same factors are present: As the count of conflicting priors underlying a particular prior rises, the value of that prior increases in importance; and the narrower the uncertainty of a prior, the more it contributes to the final solution.

Of course, limitations to our approach must be mentioned. And the key limitation is certainly that, in some practical applications, the data snooper will lack information on, either or both, best guess and uncertainty. It may be that instead, one only has upper and lower bounds for the datum of interest to inform its best guess and uncertainty. This is certainly the case in some instances when data are censored, e.g., the anti-suppression problem of Gerking et al. [13] and Isserman and Westervelt [12]. Future work using variable ranges with externally informed priors would be a natural extension of what is presented here. Indeed, some initial forays into this line of investigation are already underway, see, e.g., Makarkina and Lahr [35].

It should be mentioned that although the focus of attention here was on conflicting estimates arising from economic accounts there are other circumstances in which a formally identical problem arises, for example in expert elicitation [36].

## Figures and Tables

**Table 1:** Prior vector and concordance matrix, with no multiple estimates.

$\theta^{\prime }$	$A_{11}$	$A_{12}$	$A_{13}$	$A_{21}$	$A_{22}$	$A_{23}$	$b_{1}$	$b_{2}$	$c_{1}$	$c_{2}$	$c_{3}$	*d*
G	1	1	1	0	0	0	−1	0	0	0	0	0
	0	0	0	1	1	1	0	−1	0	0	0	0
	1	0	0	1	0	0	0	0	−1	0	0	0
	0	1	0	0	1	0	0	0	0	−1	0	0
	0	0	1	0	0	1	0	0	0	0	−1	0
	0	0	0	0	0	0	1	1	0	0	0	−1
	0	0	0	0	0	0	0	0	1	1	1	−1

**Table 2:** Prior vector and concordance matrix, with multiple estimates of $A_{23}$.

$\theta^{\prime }$	$A_{11}$	$A_{12}$	$A_{13}$	$A_{21}$	$A_{22}$	$A_{23}^{\prime }$	$b_{1}$	$b_{2}$	$c_{1}$	$c_{2}$	$c_{3}$	*d*	$A_{23}^{\prime \prime }$
G	1	1	1	0	0	0	−1	0	0	0	0	0	0
	0	0	0	1	1	1	0	−1	0	0	0	0	0
	1	0	0	1	0	0	0	0	−1	0	0	0	0
	0	1	0	0	1	0	0	0	0	−1	0	0	0
	0	0	1	0	0	0	0	0	0	0	−1	0	1
	0	0	0	0	0	0	1	1	0	0	0	−1	0
	0	0	0	0	0	0	0	0	1	1	1	−1	0
	0	0	0	0	0	1	0	0	0	0	0	0	−1

**Table 3:** Prior vector and concordance matrix, with multiple estimates of *d*.

$\theta^{\prime }$	$A_{11}$	$A_{12}$	$A_{13}$	$A_{21}$	$A_{22}$	$A_{23}$	$b_{1}$	$b_{2}$	$c_{1}$	$c_{2}$	$c_{3}$	$d^{\prime }$	$d^{\prime \prime }$
G	1	1	1	0	0	0	−1	0	0	0	0	0	0
	0	0	0	1	1	1	0	−1	0	0	0	0	0
	1	0	0	1	0	0	0	0	−1	0	0	0	0
	0	1	0	0	1	0	0	0	0	−1	0	0	0
	0	0	1	0	0	1	0	0	0	0	−1	0	0
	0	0	0	0	0	0	1	1	0	0	0	−1	0
	0	0	0	0	0	0	0	0	1	1	1	0	−1
	0	0	0	0	0	0	0	0	0	0	0	1	−1

**Table 4:** Prior vector and concordance matrix, with multiple estimates of $b_{1}$.

$\theta^{\prime }$	$A_{11}$	$A_{12}$	$A_{13}$	$A_{21}$	$A_{22}$	$A_{23}$	$b_{1}^{\prime }$	$b_{2}$	$c_{1}$	$c_{2}$	$c_{3}$	*d*	$b_{1}^{\prime \prime }$
G	1	1	1	0	0	0	−1	0	0	0	0	0	0
	0	0	0	1	1	1	0	−1	0	0	0	0	0
	1	0	0	1	0	0	0	0	−1	0	0	0	0
	0	1	0	0	1	0	0	0	0	−1	0	0	0
	0	0	1	0	0	1	0	0	0	0	−1	0	0
	0	0	0	0	0	0	0	1	0	0	0	−1	1
	0	0	0	0	0	0	0	0	1	1	1	−1	0
	0	0	0	0	0	0	1	0	0	0	0	0	−1
